# Preparation and Performance Evaluation of a Supramolecular Gel Plugging Agent for Severe Lost Circulation Gas Reservoirs

**DOI:** 10.3390/gels12030256

**Published:** 2026-03-18

**Authors:** Yingbiao Liu, Kecheng Liu, Tao Zeng, Xuyang Yao, Weiju Wang, Huijun Hao, Zhangkun Ren, Jingbin Yang

**Affiliations:** 1 Oil Production Technology Research Institute of PetroChina Xinjiang Oilfield Company, Karamay 834000, China; 2 School of Petroleum Engineering, China University of Petroleum (East China), Qingdao 266580, China; 3 CNPC Engineering Technology R&D Company Limited, China National Petroleum Corporation, Beijing 102206, China

**Keywords:** supramolecular gel, severe lost circulation, lost circulation control, rheology, swelling behavior

## Abstract

The plugging of fractured gas reservoirs with severe lost circulation during oil and gas drilling and production has long been challenged by technical issues such as low plugging strength and short effective duration. This paper reports the preparation of a high-strength supramolecular gel plugging agent via micellar copolymerization based on the synergistic effects of hydrophobic association and hydrogen bonding. Systematic optimization determined the optimal synthesis formula: acrylamide (AM) 12%, 2-acrylamido-2-methylpropanesulfonic acid (AMPS) 2%, stearyl methacrylate (SMA) 0.4%, sodium dodecyl sulfate (SDS) 1.5%, and potassium persulfate 0.3%, with a reaction temperature of 60 °C. Performance evaluations revealed that the gel possesses a controllable gelation time (120 min) and excellent viscoelastic recovery properties. At a compressive strain of 87%, the compressive stress reached 1.43 MPa while maintaining structural integrity. Swelling behavior analysis indicated that the gel follows a non-Fickian diffusion mechanism, with its swelling process governed by the synergistic interplay of water molecule diffusion and polymer network relaxation. Core plugging experiments demonstrated that the gel achieved plugging efficiencies exceeding 95% for cores with permeabilities ranging from 0.18 to 0.90 μm^2^, with a maximum breakthrough pressure gradient of up to 11.48 MPa/m. These results highlight the gel’s efficient and broad-spectrum plugging capability for fractured lost circulation zones. This preliminary study provides experimental foundations for the material design and performance optimization of supramolecular gel-based long-lasting plugging agents for severe lost circulation gas reservoirs, and further field-scale validation is required for engineering application.

## 1. Introduction

During oil and gas drilling and production, drilling fluid loss is a common and costly engineering challenge, particularly acute in fractured, heterogeneous gas zones prone to severe lost circulation [1,2,3]. Lost circulation not only leads to significant waste of materials like drilling mud, delays timelines, and substantially increases operational costs, but it may also trigger a series of serious downhole complications such as wellbore instability, stuck pipe, and even blowouts, severely hindering the safe and efficient development of hydrocarbon resources [4,5]. Therefore, developing efficient and long-lasting plugging technologies to achieve precise sealing of loss channels is crucial for ensuring drilling safety and enhancing recovery rates.

To address lost circulation, various plugging materials and technologies have been continuously developed. Early methods primarily relied on inert particulate materials (e.g., walnut shells, mica) or mechanical bridge plugs, whose mechanism is based on physical bridging and filling [6,7]. However, their effectiveness is limited for micro-fractures and seepage-type losses. With advancements in chemical technology, chemical plugging agents have gained attention due to their pumpability, ability to penetrate finer channels, and potential to form high-strength sealing bodies through chemical reactions. These have evolved into a diversified system including polymer gels, curable resins, inorganic precipitating salts, and smart responsive particles [8,9,10,11,12,13,14]. Through different mechanisms (e.g., gelation, curing, precipitation), these chemical plugging agents have demonstrated significant advantages in sealing high-permeability and fractured loss zones [15,16,17,18,19].

Gel-based plugging agents are considered one of the most promising lost circulation materials (LCMs) due to their controllable gelation time, high plugging strength, and good compatibility with formations. They are applicable to reservoirs with a wide range of permeabilities [18,20,21]. Researchers worldwide have conducted extensive innovative work in this field. Addressing lost circulation in oil-based drilling fluids for fractured formations, Bai et al. synthesized an oil-absorbing gel with both oil-absorption and plugging capabilities using butyl acrylate and sodium p-styrenesulfonate as monomers [22]. This gel featured a dense porous surface structure, achieved an oil absorption capacity of 20.93 g/g at 140 °C, and withstood a pressure of 7.6 MPa in a 3 mm wide fracture. Liu et al. prepared nano-microscale polymer gel microsphere plugging agents (AMN) via inverse emulsion polymerization [23]. These microspheres exhibited good thermal stability; adding 3% AMN to an oil-based drilling fluid reduced the high-temperature high-pressure (HTHP) fluid loss by 42% at 130 °C. He et al. fabricated a polyacrylic acid/polyacrylamide (PAA/PAM) double-network gel via solution polymerization [24]. The gel achieved a water-swelling ratio of up to 8 times and could control drilling fluid loss rates below 5 mL/min under conditions of 130 °C and 6 MPa. Studies showed this gel possesses good temperature and salt tolerance, suitable for plugging operations in high-temperature high-pressure formations. Furthermore, Qu et al. developed a self-healing gel plugging agent (PCAA) using chitosan, acrylamide, and acrylic acid as monomers [25]. The gel exhibited a self-healing efficiency of 78.5% and could effectively seal sandpack layers even at 180 °C in 8% NaCl brine. PCAA, through synergistic effects with clay particles, enhanced the rheological properties and fluid loss control capability of drilling fluids, making it suitable for high-temperature high-salinity environments.

In recent years, supramolecular gels based on non-covalent interactions have shown great potential in drilling applications. Their unique dynamically reversible network structures (e.g., hydrogen bonding, hydrophobic association, host-guest interactions) endow the materials with excellent self-healing properties, environmental stimulus responsiveness, and outstanding mechanical toughness. These characteristics align well with the inherent requirements for downhole plugging materials, which need to recover their structure after shearing during injection, remain stable in high-temperature and high-salinity environments, and resist fracturing under stress. Therefore, the supramolecular gel plugging agent is anticipated to break through the technical bottlenecks of conventional gels, thereby providing a feasible solution for tackling severe lost circulation challenges. Despite significant advancements in plugging materials based on supramolecular gels, several critical research gaps remain unresolved. On one hand, traditional covalently crosslinked polymer gels possess high mechanical strength, yet they suffer from irreversible network damage due to high shear forces during injection and lack self-healing capability once fractured downhole. On the other hand, existing supramolecular gel systems reported in the literature are primarily focused on water shut-off applications in reservoirs, with limited systematic investigations into their suitability under gas reservoir conditions characterized by high differential pressure and the risk of gas channeling. Furthermore, current research lacks a comprehensive elucidation of the mechanical strengthening mechanisms of supramolecular gels, as well as their long-term stability across a wide range of temperatures and salinities. The broad-spectrum plugging efficiency of these gels for fractures of varying scales also requires further in-depth validation.

Based on this, this paper aims to conduct a preliminary experimental investigation on the development of a high-strength supramolecular gel suitable for plugging severe lost circulation gas zones. The research employs a micellar copolymerization method, using acrylamide, 2-acrylamido-2-methylpropanesulfonic acid, and stearyl methacrylate as the main monomers. By optimizing the ratio between hydrophobic association and hydrophilic components, a gel system with a dynamically reversible network was prepared. This study systematically investigated the effects of monomer ratio, initiator, and temperature on the gelation behavior, determining the optimal synthesis formula and process. A comprehensive laboratory evaluation of its rheological properties, swelling behavior, mechanical strength, and core plugging efficacy was conducted, and its plugging mechanism was explored in depth. This work represents a foundational, laboratory-scale investigation focused on material synthesis, mechanism elucidation, and initial performance screening. The findings provide an experimental basis and theoretical reference for the future optimization and potential engineering application of supramolecular gel lost circulation materials.

## 2. Results and Discussion

### 2.1. Preparation and Optimization of the Supramolecular Gel

#### 2.1.1. Effect of Polymer Monomers on the Supramolecular Gel

**Figure 1:**
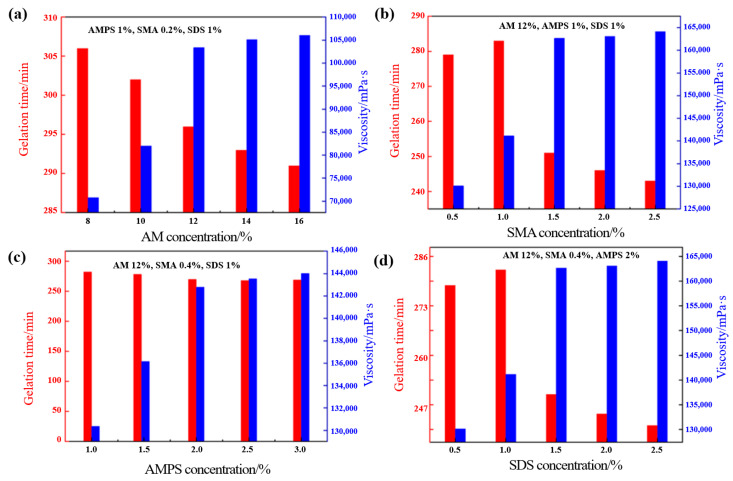
Effects of different monomer concentrations on the gelation time and viscosity of the supramolecular gel: (**a**) AM; (**b**) SMA; (**c**) AMPS; (**d**) SDS.

Using a single-factor experimental method, the effects of various monomer ratios on the gelation behavior and rheological properties of the supramolecular gel were systematically studied, as shown in Figure 1. Figure 1a shows that as the AM concentration increased, the system’s gelation time gradually shortened, and the apparent viscosity increased accordingly. When the AM concentration reached 12%, both the gelation time and viscosity variation tended to stabilize, indicating that the polymer backbone network had essentially formed at this stage, and further increases in monomer concentration yielded limited improvements in the gel structure. This trend is consistent with the findings reported by Bai et al. [22]. At relatively low monomer concentrations, increasing the concentration accelerated the polymerization rate, extended the polymer chain length, and enhanced the crosslinking density of the network, manifesting as a shortened gelation time and increased viscosity. However, beyond a certain threshold concentration, although the collision probability among monomers further increased, excessive concentration exacerbated chain transfer reactions and caused a sharp rise in system viscosity, which hindered the diffusion of free radicals and adversely affected the uniformity of the network structure. Consequently, the enhancement in gel performance plateaued or even declined. Therefore, the optimal AM concentration was determined to be 12%. Figure 1b indicates that the gel viscosity reached its maximum when the SMA concentration was 0.4%. Beyond this concentration, viscosity growth was not significant, suggesting that the number of hydrophobic association points approached saturation. Excessive hydrophobic groups might instead cause local aggregation, affecting network uniformity. Figure 1c shows that the system exhibited the fastest gelation rate and stable viscosity at an AMPS concentration of 2%. Increasing the AMPS dosage further provided limited improvement in gel viscosity, possibly due to enhanced electrostatic repulsion between sulfonate groups [21]. For the surfactant SDS (Figure 1d), the gel viscosity peaked at a concentration of 1.5%, beyond which viscosity no longer increased or even slightly decreased. This phenomenon can be attributed to the dual role of SDS in micellar copolymerization: at appropriate concentrations, it effectively solubilizes hydrophobic monomers and stabilizes the micellar structure, promoting copolymerization; however, excessive concentrations may disrupt the orderly assembly of polymer chains or even cause changes in micelle morphology, thereby affecting the integrity and strength of the final gel network [11]. Based on these results, the optimal synthesis formula for the supramolecular gel was determined as: AM 12%, AMPS 2%, SMA 0.4%, SDS 1.5%.

#### 2.1.2. Effect of Initiator on the Supramolecular Gel

**Figure 2:**
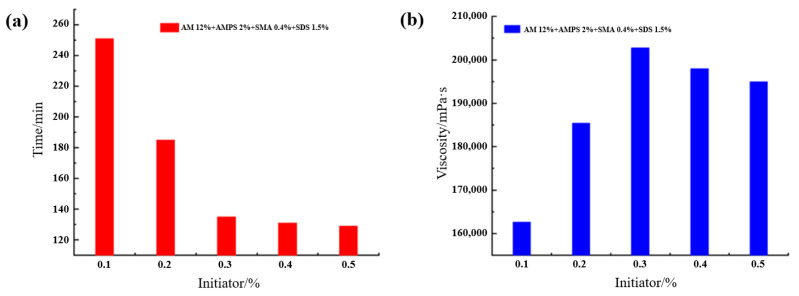
Effect of initiator on the gelation time and viscosity of the supramolecular gel plugging agent: (**a**) gelation time; (**b**) gel viscosity.

This study employed a potassium persulfate/sodium bisulfite redox initiation system. This system generates free radicals via electron transfer reactions, significantly lowering the activation energy and enabling polymerization initiation at lower temperatures. However, in high-temperature formation environments, excessively rapid initiator decomposition may lead to premature consumption of free radicals, affecting the full formation of the gel network. To clarify the influence mechanism of initiator dosage on gelation behavior, experiments systematically investigated the evolution of gelation time and viscosity under different initiator amounts. As shown in Figure 2, as the initiator amount increased, the gelation time of the supramolecular gel gradually shortened, while the viscosity showed a trend of first increasing and then decreasing. When the initiator dosage was 0.3%, the system completed gelation within 135 min and the maximum viscosity exceeded 200,000 mPa·s. This phenomenon can be attributed to the synergistic effect of initiator decomposition kinetics and free radical concentration: an appropriate amount of initiator provides a suitable concentration of primary free radicals, promoting monomer polymerization and hydrophobic chain association, thereby forming a dense and uniform three-dimensional network. However, excessive initiator leads to overly rapid free radical generation, potentially intensifying chain termination reactions, broadening the polymer molecular weight distribution, reducing the uniformity of network crosslinking, and consequently weakening the mechanical properties and structural stability of the gel. These results indicate that gelation kinetics can be effectively regulated by controlling the initiator dosage, providing a basis for formula design to adapt to different formation temperature conditions for lost circulation operations.

#### 2.1.3. Effect of Synthesis Temperature on the Supramolecular Gel

**Figure 3:**
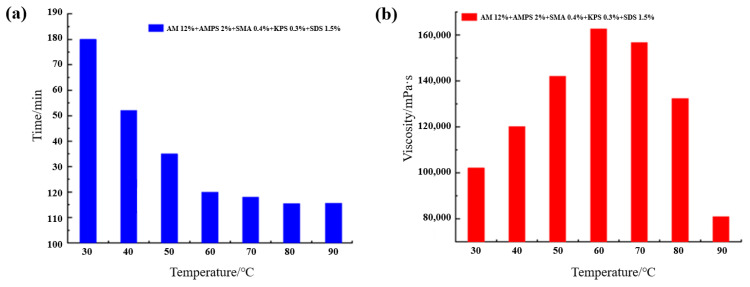
Effect of temperature on the gelation time and viscosity of the supramolecular gel plugging agent: (**a**) gelation time; (**b**) gel viscosity.

Based on the determined optimal formula, the effects of reaction temperature (30–90 °C) on the gelation behavior and viscosity development of the supramolecular gel were further investigated, as shown in Figure 3. As the temperature increased, the gelation time progressively shortened. The decrease was significant within the 30–60 °C range and tended to level off beyond 60 °C. Meanwhile, the gel viscosity showed a trend of first increasing and then decreasing with temperature, reaching a peak at 60 °C, corresponding to a gelation time of 120 min. This phenomenon can be explained from both polymerization kinetics and network structure stability perspectives: moderate heating accelerates initiator decomposition, increases monomer activity, promotes micelle formation and hydrophobic association, which is beneficial for constructing a uniform and dense network structure. However, excessively high temperatures (>60 °C) may intensify chain transfer reactions, disrupt micelle stability, or even cause dissociation of hydrophobic association points, leading to increased network structure defects, macroscopically manifested as viscosity decrease and performance degradation [26]. Therefore, 60 °C is the optimal reaction temperature for this system, balancing reaction efficiency and gel structural integrity.

In summary, a supramolecular gel with synergistic hydrophobic association and hydrogen bonding was successfully prepared via micellar copolymerization. The optimal reaction temperature for the system is 60 °C, and the optimized synthesis formula is: AM 12%, AMPS 2%, SMA 0.4%, SDS 1.5%, initiator 0.3%. This gel exhibits good controllable gelation and structural stability, possessing the potential to serve as a high-performance plugging material for severe lost circulation gas reservoirs. To further clarify its plugging mechanism and promote field application, subsequent systematic evaluations of the gel’s physicochemical properties and plugging efficiency were conducted.

### 2.2. Characterization of the Supramolecular Gel

**Figure 4:**
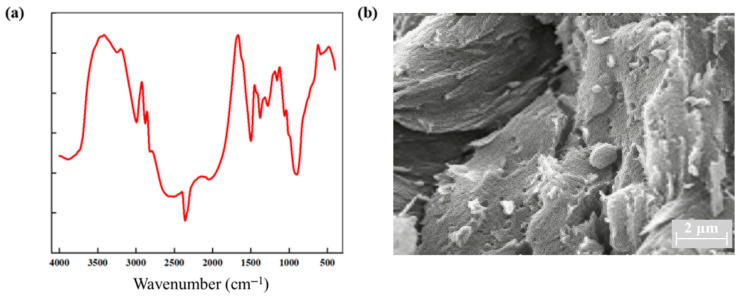
Structural characterization of the supramolecular gel: (**a**) FTIR; (**b**) SEM.

The Fourier transform infrared spectroscopy (FTIR) in Figure 4a shows broad and strong absorption peaks at 3412 cm^−1^ and 3197 cm^−1^, attributed to N–H and O–H stretching vibrations, respectively. The significant broadening and shift towards lower wavenumbers indicate the presence of a strong intermolecular hydrogen bonding network within the system, possibly accompanied by the coexistence of multiple hydrogen bonding modes [10]. A sharp C=O stretching vibration peak observed at 1676 cm^−1^ further confirms the presence of amide groups. Its slight shift compared to pure monomers suggests that C=O also participates in hydrogen bond formation [27]. These features collectively indicate that the polymer chains interact through multiple hydrogen bonds, constructing a stable three-dimensional network structure. The scanning electron microscopy (SEM) images (Figure 4b) reveal that the gel possesses a clear layered interconnected morphology. The layers have uniform thickness and are tightly connected by fibrous structures, forming a continuous and dense hierarchical network. This structure facilitates stress transfer and dissipation and corroborates the regulatory effect of synergistic hydrophobic association and hydrogen bonding on the gel’s microstructure. Both FTIR and SEM results confirm the successful synthesis of the supramolecular gel, with significant hydrogen bonding and hydrophobic association interactions present within the system, laying a structural foundation for its macroscopic mechanical properties and plugging efficiency.

### 2.3. Rheological Properties of the Supramolecular Gel

**Figure 5:**
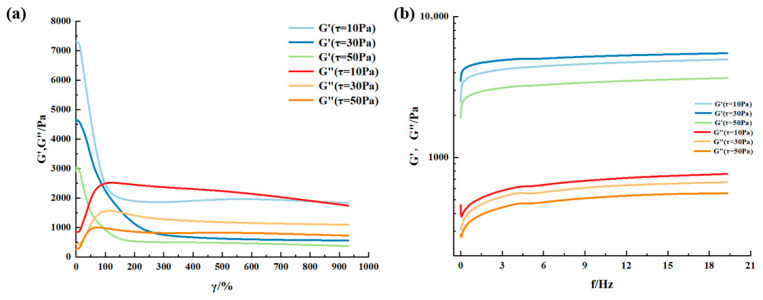
Rheological behavior of supramolecular gels at room temperature (20 °C): (**a**) modulus versus shear strain at constant shear stress; (**b**) modulus versus shear frequency at constant shear stress.

**Figure 6:**
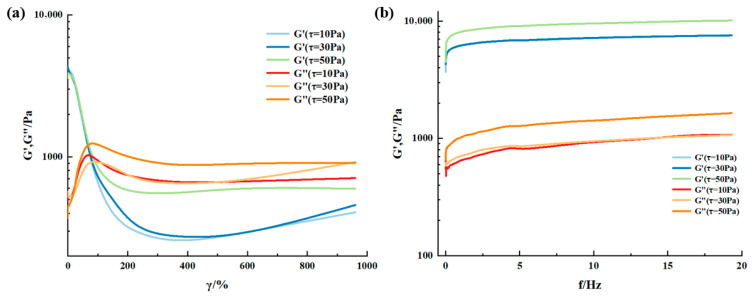
Rheological behavior of supramolecular gels at 60 °C: (**a**) modulus versus shear strain at constant shear stress; (**b**) modulus versus shear frequency at constant shear stress.

Most viscoelastic materials are subjected to varying degrees of stress in specific applications. For any given application, there is always a certain shear limit, and optimal rheological properties must be maintained according to the desired outcome. Therefore, materials need to maintain homogeneity and preserve their structure under high shear. Rheology is one technique for characterizing viscoelastic material properties. The variation of moduli with shear strain and frequency is a common tool for characterizing and comparing material rheological behavior. To determine the rheology of the supramolecular gel, shear strain and frequency sweep tests were performed on gel samples at different temperatures using a HAAKE MARS 60 high-temperature high-pressure rotational rheometer. The experimental results are shown in Figure 5 and Figure 6. The research findings indicate that changes in shear strain and frequency affect the rheology of the supramolecular gel. When the shear strain exceeds 80%, the storage modulus (G′) of the gel decreases significantly, indicating the onset of network disruption and a sol–gel phase transition. Before this transition point, G′ remains stable at approximately 1000 Pa, and the loss modulus (G″) is low, indicating that the gel exhibits solid-like elastic behavior under small strains, effectively resisting scouring by formation fluids. Frequency sweep results show that within the 0–20 Hz range, both G′ and G″ increase with frequency, and G′ is consistently greater than G″, confirming that the gel can maintain its network structure under dynamic shear, possessing good structural stability and energy dissipation capacity [28]. Comparing rheological behavior at different temperatures reveals that the modulus values of G′ and G″ at 60 °C are higher than those at 20 °C overall, indicating that temperature also influences its viscoelasticity. The higher the temperature, the greater the storage and loss moduli, with a corresponding weakening of elastic capability. A similar situation was observed in Wang et al.’s research [29]. They suggested that with increasing temperature and frequency, the large molecular chains within the gel sample struggle to rearrange into a regular network structure, causing the gel to dehydrate and harden under heating and shear, losing its sol–gel phase transition ability, leading to a weakening of its elastic capability. This characteristic provides guidance for balancing injection and retention control of gels in high-temperature formations.

**Figure 7:**
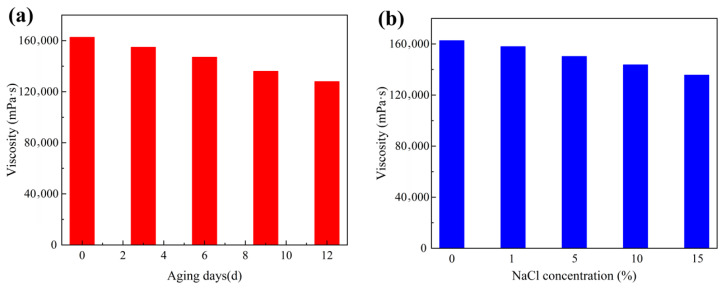
Temperature and salt resistance performance of the supramolecular gel: (**a**) viscosity variation after different aging periods at 120 °C; (**b**) viscosity variation under different NaCl concentrations.

To comprehensively evaluate the applicability of this supramolecular gel under realistic formation conditions, its long-term stability was investigated over 12 days in high-salinity (15% NaCl) and high-temperature (120 °C) environments. As shown in Figure 7a, after 12 days of thermal aging, the viscosity of the supramolecular gel decreased from an initial value of 162,663 mPa·s to 128,352 mPa·s, representing a reduction of approximately 21.1%. Despite this decrease, the gel retained a high absolute viscosity (>1.2 × 10^5^ mPa·s), indicating that its network structure did not undergo catastrophic failure under high-temperature conditions. This phenomenon can be attributed to the dynamic reversibility of hydrophobic associations within the supramolecular gel. At elevated temperatures, partial dissociation of hydrophobic association sites occurs temporarily, leading to a slight reduction in crosslinking density and consequently a decrease in viscosity. However, owing to the thermally reversible nature of the physical crosslinks formed between hydrophobic segments and surfactant micelles, partially dissociated chains can reorganize and form new associative structures during static aging, thereby partially compensating for the loss in network strength. As illustrated in Figure 7b, after aging for 12 days in a high-salinity environment containing 15% NaCl, the viscosity of the supramolecular gel decreased to 135,674 mPa·s, corresponding to a reduction of approximately 16.6%, which is slightly lower than that observed under the sole influence of high temperature. This result demonstrates the excellent salt tolerance of the gel, as its network structure maintains considerable integrity even under high-salinity conditions. This outstanding performance is primarily attributed to the sulfonate groups present in the AMPS monomer incorporated into the polymer chains. These sulfonate groups possess strong hydration capacity and excellent anti-salting-out ability. Even under the pronounced charge screening effect of Na^+^ ions, they maintain the hydration layer surrounding the polymer chains through their strong hydrophilicity, thereby inhibiting network collapse.

### 2.4. Swelling Properties of the Supramolecular Gel

**Figure 8:**
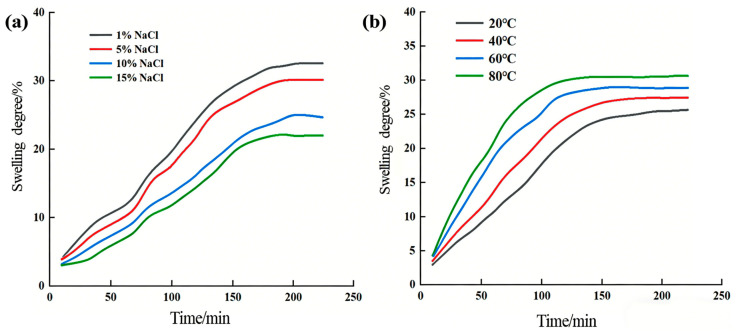
Effects of (**a**) NaCl solution concentration and (**b**) temperature on the swelling performance of the supramolecular gel.

Figure 8 illustrates the variation patterns of swelling behavior of the supramolecular gel under different salt concentrations and temperatures. Regarding the influence of salt concentration (Figure 8a), the equilibrium swelling degree of the gel in 1% NaCl solution (32.5%) was significantly higher than that in 15% NaCl solution (22.1%). This is primarily attributed to the charge shielding effect of salt ions on the gel network: higher salt concentrations compress the double layer around polymer chains, weakening the electrostatic repulsion between ionic groups (e.g., sulfonate groups from AMPS), leading to network contraction and reduced swelling capacity [30]. Additionally, the decreased osmotic pressure difference caused by external salt ions further inhibits water molecule penetration. Regarding the influence of temperature (Figure 8b), as the temperature increased from 30 °C to 60 °C, the equilibrium swelling degree of the gel gradually increased. This is because increasing temperature enhances the diffusion coefficient of water molecules and increases the mobility of polymer chain segments, making the network easier to expand and accommodate more water molecules. However, as the temperature continues to rise, hydrophobic association may weaken, some physical crosslinking points may dissociate, affecting network stability, and the increase in swelling degree tends to level off. Overall, the swelling behavior of the supramolecular gel is synergistically regulated by salt concentration and temperature. Its swelling process is jointly determined by water molecule diffusion rate and polymer chain relaxation kinetics, exhibiting typical non-Fickian diffusion characteristics. This property is of significant importance for the adaptive plugging behavior of gels in complex formation environments.

To further elucidate the influence mechanisms of temperature and salt concentration on the gel swelling process, a quantitative analysis of its swelling kinetics was conducted. The following power-law model was used to fit the swelling data:

(1) $$S_{R}/S_{eq}=K_{s}t^{n}$$

wherein *K_s_* is the characteristic rate constant of the gel, and *n* is the diffusion exponent, used to determine the type of swelling mechanism. Typically, *n* ≤ 0.5 is classified as Fickian diffusion (diffusion-controlled), 0.5 < *n* < 1.0 as non-Fickian diffusion (coupled diffusion and relaxation), and *n* ≥ 1.0 as relaxation-controlled.

**Figure 9:**
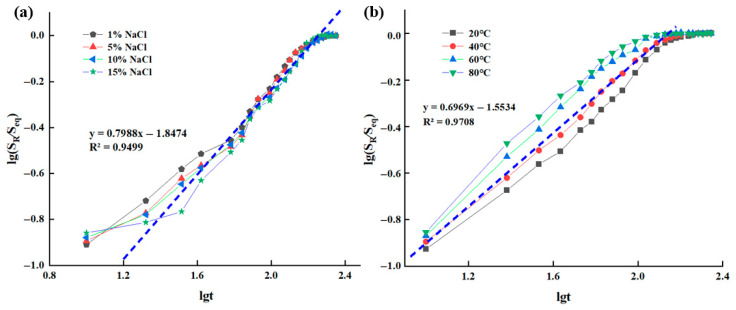
Swelling kinetics fitting curves of supramolecular gels: (**a**) swelling fitting curves at different NaCl salt concentrations; (**b**) swelling fitting curves at different temperatures.

Logarithmic linear fitting of the experimental data (Figure 9) yielded the kinetic parameters listed in Table 1. Under different salt concentrations and temperatures, the diffusion exponent n consistently fell between 0.5 and 1.0, indicating that the gel’s swelling process consistently exhibited a non-Fickian diffusion mechanism. This means the penetration rate of water molecules and the relaxation rate of polymer network segments are on the same time scale. During swelling, the gel network can dynamically adjust to gradually adapt to solvent penetration, rather than undergoing instantaneous swelling or structural disruption.

### 2.5. Mechanical Properties of the Supramolecular Gel

**Figure 10:**
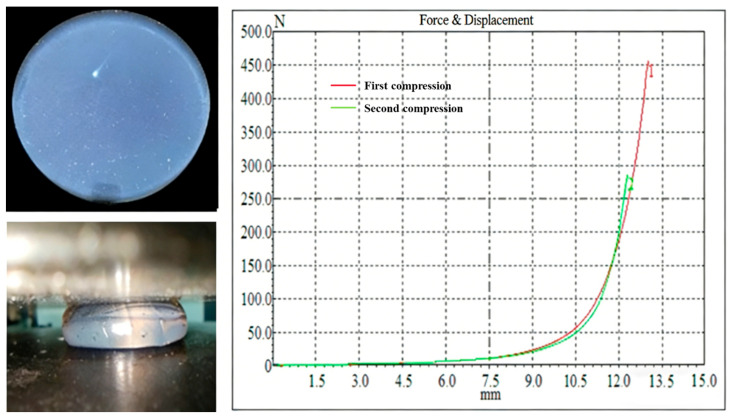
Compressive properties of supramolecular gels.

Uniaxial compression tests were performed on the supramolecular gel using an electronic universal testing machine, with results shown in Figure 10. The gel exhibited good mechanical response during compression: when the compression displacement reached 13 mm (corresponding to a compressive strain of approximately 87%), the compressive stress was 1.43 MPa, and no obvious cracks appeared on the sample surface, indicating excellent compressive resistance and structural integrity. This mechanical behavior is primarily attributed to the design of the dynamic crosslinked network within the gel. The long straight-chain alkyl structure in the hydrophobic monomer SMA is similar to the hydrophobic tail structure of the surfactant SDS, allowing it to be effectively solubilized and uniformly distributed within micelles during micellar copolymerization, forming physical crosslinking points with moderate density and uniform distribution [21]. Under compressive load, these hydrophobic association points can dissipate energy through reversible dissociation–recombination mechanisms while maintaining overall network continuity, thereby endowing the gel with high compressive strength and good toughness. Furthermore, the synergistic enhancement effects of non-covalent interactions like hydrogen bonds further improve the energy dissipation capacity and structural stability of the gel network, allowing it to remain intact even under large deformations.

### 2.6. Compatibility of the Supramolecular Gel with Drilling Fluid

**Figure 11:**
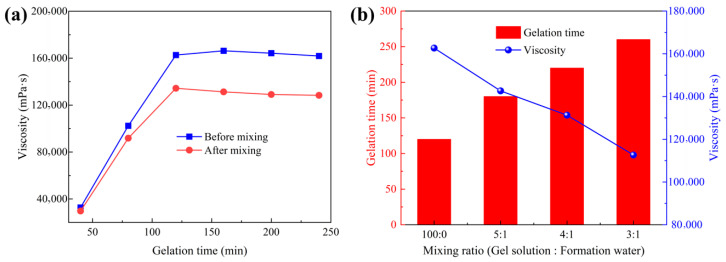
Effect of base drilling fluid on the gelation performance of the supramolecular gel: (**a**) variation of gel viscosity with gelation time before and after mixing with base drilling fluid; (**b**) effect of simulated formation water on gelation time and viscosity of gel solution.

To evaluate the applicability of the supramolecular gel in actual drilling operations, its compatibility with the base drilling fluid and its gelation behavior under formation water contamination were further investigated. A 4.0% bentonite base slurry (density 1.05 g·cm^−3^) was mixed with the gel solution at an equal volume ratio, and the flocculation state and rheological property variations in the mixed system were observed, with results presented in Figure 11a. Compared with the pure gel solution, the viscosity of the mixed system exhibited a certain degree of decrease, with a reduction of approximately 17.4%. This phenomenon can be primarily attributed to two factors. First, the bentonite particles in the base drilling fluid carry negative surface charges, which can physically adsorb onto the amide groups of the polymer chains, partially interfering with the hydrophobic associations among polymer chains and resulting in a slightly loosened network structure. Second, the introduction of the base slurry effectively dilutes the gel system, reducing the polymer concentration per unit volume and consequently leading to a decrease in viscosity. However, it is noteworthy that after mixing with the base slurry, the gelation time of the system remained almost unchanged, and a complete gel structure was ultimately formed. This result indicates that although bentonite particles exert some influence on the initial viscosity, the gel maintains favorable gelation performance, suggesting good compatibility between the supramolecular gel and the base drilling fluid. The gel’s resistance to formation water contamination was further investigated, with results shown in Figure 11b. The ionic composition of the simulated formation water is presented in Table 2. As illustrated in Figure 11b, with a gradual increase in the mixing ratio of simulated formation water to gel solution, the gelation time exhibited a progressive prolongation, while the final gel viscosity gradually decreased. When the volume ratio of simulated formation water to gel solution reached 3:1, the viscosity retention rate of the gel was approximately 70%. This can be attributed to the charge shielding effect of high-concentration cations, which weakens the electrostatic repulsion between molecular chains, leading to network loosening; meanwhile, the dilution effect reduces the reactant concentration and delays the gelation process. Nevertheless, even under high-ratio formation water contamination, the system was still capable of forming a complete gel, with a viscosity retention rate significantly superior to that of conventional polyacrylamide gels, demonstrating the excellent resistance of this supramolecular gel to formation water contamination.

### 2.7. Plugging Performance of the Supramolecular Gel

To evaluate the plugging performance of the supramolecular gel under simulated formation conditions, core plugging experiments were conducted using a high-temperature, high-pressure lost circulation displacement apparatus. The experimental results (Table 3) indicate that the gel exhibited excellent plugging capability for cores with different permeabilities (0.18–0.90 μm^2^), achieving plugging efficiencies all above 95%. The post-plugging permeability of all cores dropped below 0.02 μm^2^, indicating that the gel formed a dense, continuous plugging layer within pore throats, effectively blocking fluid channeling paths. As the initial core permeability increased, the breakthrough pressure of the gel gradually rose from 235.8 kPa to 803.5 kPa, corresponding to breakthrough pressure gradients ranging from 3.37 to 11.48 MPa/m. This trend reflects that in high-permeability channels, the gel precursor solution can more fully enter the pore space and form a thicker, more structurally complete plugging slug through in situ gelation, thereby possessing higher pressure resistance. This characteristic indicates that the supramolecular gel is particularly suitable for plugging severe lost circulation channels in fractured or high-permeability zones, offering significant advantages in maintaining wellbore stability and controlling drilling fluid loss.

**Figure 12:**
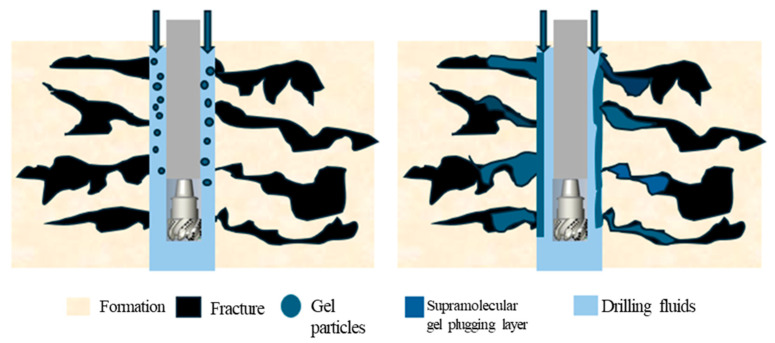
Schematic diagram of the plugging mechanism of the supramolecular gel.

Figure 12 further elucidates the lost circulation control mechanism of the supramolecular gel. Unlike traditional covalently crosslinked gels, supramolecular gels self-assemble through dynamic non-covalent interactions such as hydrogen bonds, hydrophobic associations, and electrostatic interactions, endowing their network structure with reversibility and environmental responsiveness. The dynamic equilibrium of these weak interactions allows the gel to re-heal after shear disruption and maintain structural stability under changing external conditions like temperature and salinity. As a plugging material, leveraging its excellent viscoelasticity and adaptive capability, this gel can enter formation micro-fractures and pore throats during injection. Efficient plugging is achieved through physical adsorption and bridging, dynamic network reconstruction, and increased flow resistance. Additionally, ionic groups in the gel (e.g., sulfonate provided by AMPS) maintain the network in an expanded state via electrostatic repulsion while ensuring structural stability in saline solutions through charge shielding effects, conferring good temperature and salt tolerance to the material. This multi-mechanism synergistic plugging approach makes the supramolecular gel especially suitable for highly heterogeneous, severely lost-circulation fractured formations.

## 3. Conclusions

This study prepared a high-strength supramolecular gel plugging agent based on synergistic hydrophobic association and hydrogen bonding. Its synthesis, structure, properties, and plugging efficiency were systematically evaluated. The main conclusions are as follows:

(1) The optimal synthesis conditions for the supramolecular gel are: AM 12%, AMPS 2%, SMA 0.4%, SDS 1.5%, initiator 0.3%, and reaction temperature of 60 °C. Under these conditions, the gelation time is 120 min, exhibiting good pumpability and controllable gelation characteristics.
(2) The gel demonstrates excellent viscoelastic properties and mechanical strength: under dynamic shear, the storage modulus is higher than the loss modulus, indicating strong structural recovery capability; at a uniaxial compressive strain of 87%, the stress is 1.43 MPa, with good network integrity, showing high compressive and shear deformation resistance.
(3) Swelling behavior studies show that the gel follows a non-Fickian diffusion mechanism in different salinity and temperature environments. Its swelling process is jointly controlled by water molecule penetration and polymer chain segment relaxation, reflecting good environmental adaptability and structural stability.
(4) Core plugging evaluation results indicate that the gel achieves plugging efficiencies exceeding 95% for cores with permeabilities ranging from 0.18 to 0.90 μm^2^, with breakthrough pressure gradients reaching 3.37–11.48 MPa/m. This proves it can effectively plug fractures and high-permeability channels, making it suitable for high-strength, long-lasting plugging of severe lost circulation gas reservoirs.

Despite the promising results demonstrated in this laboratory study, several limitations should be acknowledged. Firstly, the plugging performance was evaluated using artificial cores with uniform permeability under static conditions. This does not fully replicate the complex downhole environment characterized by dynamic fracture width variations, fluid erosion, and coupled temperature-pressure fluctuations. Secondly, while the gel exhibits good thermal stability over 12 days, the long-term mechanical integrity and chemical stability over extended operational periods remain to be validated. Thirdly, the scalability of the synthesis process and the economic feasibility for large-scale field applications have not been assessed. Therefore, while this study confirms the potential of the supramolecular gel as a plugging agent, further research involving simulated formation damage tests, long-term dynamic aging, and pilot-scale field trials is necessary to translate these laboratory findings into a mature technology for severe lost circulation control.

## 4. Materials and Methods

### 4.1. Materials and Instruments

Acrylamide (AM, AR, 99%) and 2-acrylamido-2-methylpropanesulfonic acid (AMPS, AR, 98%) were sourced from Shanghai Macklin Biochemical Co., Ltd., Shanghai, China. Stearyl methacrylate (SMA, AR, 96%), sodium dodecyl sulfate (SDS, AR, 99%), and sodium bisulfite (AR, 99%) were obtained from Shanghai Aladdin Biochemical Technology Co., Ltd., Shanghai, China. Anhydrous sodium sulfate (SSA, AR, 99%), sodium chloride (NaCl, AR, 99.8%), and potassium persulfate (KPS, AR, 99.9%) were provided by Sinopharm Chemical Reagent Co., Ltd., Shanghai, China.

### 4.2. Preparation of the Supramolecular Gel

Taking the preparation of 100 g of supramolecular gel solution as an example, five sample groups were prepared. For each group, 85 g of deionized water was weighed into a beaker. Sequentially, 12 g of AM, 2 g of AMPS, and 0.4 g of SMA were added, along with SDS in five gradients: 0.5 g, 1.0 g, 1.5 g, 2.0 g, and 2.5 g. During preparation, AM and AMPS were first dissolved in deionized water, followed by the corresponding amount of SDS, and stirred at 200 rpm until a homogeneous system formed. Subsequently, SMA was added, the stirring speed was increased to 400 rpm, and after mixing, the initiator was added with continuous stirring for 1 h. The entire process was conducted under an inert atmosphere (nitrogen). After rapid degassing, the mixture was transferred to a reaction vessel. Finally, the vessel was placed in an oven at a constant temperature of 60 °C to complete the gel preparation.

### 4.3. Characterization of the Supramolecular Gel

(1) FTIR Analysis

The chemical structure of the supramolecular gel was characterized using an FTIR-7600 Fourier Transform Infrared Spectrometer (Shanghai Precision Instruments Co., Ltd., Shanghai, China). The dried and ground sample was mixed with potassium bromide and pressed into a pellet. Scanning was performed in the wavelength range of 4000–400 cm^−1^ to analyze characteristic absorption peaks for identifying functional groups and intermolecular interactions.

(2) SEM Analysis

A Quanta200F field emission scanning electron microscope (Thermo Fisher, Waltham, MA, USA) was used to observe the microstructure of the gel. The freeze-dried sample surface was sputter-coated with gold and observed under high vacuum at an accelerating voltage of 5 kV to examine its surface and internal cross-sectional morphology.

### 4.4. Rheological Performance Evaluation of the Supramolecular Gel

The viscoelasticity of the gel was evaluated using a HAAKE MARS 60 high-temperature high-pressure rotational rheometer (Thermo Fisher, Waltham, MA, USA). Strain sweep tests (from 0.1% to 1000%) and frequency sweep tests (from 0 to 20 Hz) were conducted at 20 °C and 60 °C, respectively. The storage modulus and loss modulus were recorded as functions of strain and frequency.

### 4.5. Compression Performance Evaluation of the Supramolecular Gel

The supramolecular gel prepared with the optimized formula was injected into specific molds to form cylindrical specimens of uniform size (base diameter 20.0 ± 0.2 mm, height 15.0 ± 0.3 mm). After equilibrating at room temperature for 24 h, uniaxial compression tests were performed using a CMT4000 electronic universal testing machine (Jinan Xin Shi Jin Testing Machine Co., Ltd., Jinan, China). Tests were conducted at room temperature (25 ± 2 °C) with a compression rate set at 3 mm/min, corresponding to an initial strain rate of approximately 0.0033 s^−1^. During the experiment, the upper platen moved downward at a constant speed, and load and displacement data were continuously recorded and converted into engineering stress–strain curves.

### 4.6. High-Temperature High-Pressure Displacement Experiment

Three groups of artificial sandstone cores (labeled BY-1, BY-2, BY-3) with uniform geometric dimensions (length 7.00 ± 0.05 cm, diameter 2.50 ± 0.02 cm) were selected. Prior to experiments, core porosity was measured using a porosimeter, and initial gas permeability (using nitrogen as the medium at an average pressure of 1.0 MPa) was measured via the steady-state method to establish a basic property database. Subsequently, the prepared supramolecular gel precursor solution was loaded into the intermediate container of an XS-PPA high-temperature high-pressure lost circulation displacement apparatus (Qingdao Xusheng Petroleum Instrument Co., Ltd., Qingdao, China). The core was loaded into a core holder, and confining pressure was applied to simulate formation conditions. At an experimental temperature of 60 °C, the gel solution was injected into the core at a constant flow rate, with a total injection volume of 2.5 PV. The pressure change at the injection end was monitored and recorded in real-time during injection. After injection, flow was stopped, and the system was held static under constant temperature for 2 h to ensure sufficient gelation within the core. Following gelation, nitrogen was injected into the core holder for displacement at a constant flow rate, while the injection pressure was gradually increased. The pressure change was monitored in real-time. When a pressure drop or stabilization occurred, the maximum pressure value achieved during this stage was recorded as the breakthrough pressure. After all plugging experiments, the gas permeability of the cores was measured again, and the plugging efficiency was calculated.

## Figures and Tables

**Table 1:** Swelling kinetics parameters of supramolecular gels.

Influencing Factor	Fickian Characteristic Index (*n*)	Intercept	Fitting Correlation Coefficient (R^2^)
NaCl concentration	0.80	−1.85	0.950
Temperature	0.70	−1.55	0.971

**Table 2:** Ion composition of simulated formation water.

Ion Composition	Na^+^	Ca^2+^	Mg^2+^	Cl^−^	SO_4_^2−^	HCO_3_^−^	Br^−^	I^−^	Total
Concentration (mg/L)	174,566	30,473	4367	273,600	154	70	211	12	483,453

**Table 3:** Plugging performance of the supramolecular gel system.

Core	Length/cm	Diameter/cm	Porosity/%	Permeability Before Plugging/μm^2^	Permeability After Plugging/μm^2^	Plugging Rate/%	Breakthrough Pressure/kPa	Breakthrough Pressure Gradient/MPa/m
BY-1	7	2.5	18.35	0.1816	0.0083	95.43	235.8	3.37
BY-2	7	2.5	22.04	0.4237	0.0131	96.91	512.6	7.32
BY-3	7	2.5	25.78	0.9013	0.0201	97.77	803.5	11.48

## Data Availability

Data is contained within the article.
